# Preliminary study of alcohol problem severity and response to brief intervention

**DOI:** 10.1186/s13722-021-00262-6

**Published:** 2021-08-24

**Authors:** Lindsay R. Meredith, Erica N. Grodin, Mitchell P. Karno, Amanda K. Montoya, James MacKillop, Aaron C. Lim, Lara A. Ray

**Affiliations:** 1 Department of Psychology, University of California Los Angeles, 1285 Franz Hall, Box 951563, Los Angeles, CA 90095-1563 USA; 2 Department of Psychiatry and Biobehavioral Sciences, University of California, Los Angeles, Los Angeles, CA USA; 3 Department of Psychiatry and Behavioural Neurosciences, McMaster University, Hamilton, ON Canada

**Keywords:** Brief intervention, Alcohol, Problem severity, Motivation to change

## Abstract

**Background:**

Findings have been mixed as to whether brief intervention (BI) is appropriate and effective for individuals with more severe alcohol use problems. Motivation to change drinking has been supported as a mechanism of behavior change for BI. This exploratory study examined aspects of motivation as mechanisms of clinical response to BI and alcohol problem severity as a moderator of treatment effects.

**Methods:**

Non-treatment-seeking heavy drinkers (average age = 35 years; 57% male) were randomized to receive BI (n = 27) or attention-matched control (n = 24). Three indices of motivation to change were assessed at baseline and post-intervention: importance, confidence, and readiness. Moderated mediation analyses were implemented with treatment condition as the focal predictor, changes in motivation as mediator, 1-month follow-up drinks per day as the outcome, and an alcohol severity factor as second-stage moderator.

**Results:**

Analysis of importance displayed a significant effect of intervention condition on importance (*p* < 0.003) and yielded a significant index of moderated mediation (CI − 0.79, − 0.02), indicating that the conditional indirect effect of treatment condition on drinking through importance was stronger for those with higher alcohol severity. For all motivation indices, alcohol severity moderated the effect of post-intervention motivation levels on drinking (*p*’s < 0.05). The direct effect of treatment condition on drinking was not significant in any model.

**Conclusions:**

Findings highlight the relevance of considering one’s degree of alcohol problem severity in BI and alcohol screening efforts among non-treatment seeking heavy drinkers. These nuanced effects elucidate both potential mechanisms and moderators of BI response.

*Trial registration* Clinicaltrials.gov: NCT04710095. Registered January 14, 2021—retrospectively registered, https://clinicaltrials.gov/ct2/show/study/NCT04710095.

## Introduction

Heavy alcohol use is highly prevalent in the United States with recent estimates of almost 15 million individuals meeting criteria for past-year alcohol use disorder (AUD) and over a quarter of adults reporting past month binge drinking (SAMHSA, 2019). Despite the prevalence of AUD and numerous available evidenced-based psychosocial and pharmacological interventions [1], treatment rates for AUD remain low [2]. Problematic alcohol use is highly heterogenous, with distinctions in type and severity of alcohol-related harms experienced. With this variability in mind, certain treatments may be better targeted for individuals who are higher or lower on the AUD severity spectrum [1]. Brief intervention (BI) is supported as an effective behavioral strategy designed to address risky drinking in individuals with heavy alcohol use who have yet to transition to a more severe AUD profile [3]. BI typically consists of a single session, ranging from 5 to 60 min, and is designed to increase one’s motivation for behavior change (i.e., drinking reductions) by encouraging self-awareness and monitoring of high-risk drinking situations and alcohol-related consequences. Across BIs, several core components are shared and include providing feedback on normative drinking levels and individualized risk, inquiring about desire to change drinking, and collaborating on a plan to change behavior. Research in this area has consistently demonstrated reductions in drinking after BI [4–6].

Promoting motivation for behavior change by identifying reasons and need for change, is thought to be a core component of BI [7–9]. Motivation to change is a fluid, multi-dimensional construct that indicates an individual’s openness to participate in a behavior change plan (i.e., a plan with specific action steps to reach a drinking goal; [10]). It is thought to incorporate an individual’s understanding of the importance of behavior change, confidence in their ability to make a change, and readiness to make this change. Several studies have demonstrated positive associations between BI, one’s motivation, and drinking outcomes, such that enhancing motivation may serve as an important mechanism for behavior change [11–14]. For example, among chronically homeless individuals, post-intervention motivation to change was associated with decreases in alcohol use 2 years later [11]. In adolescents and young adults, greater readiness to change was positively related to clinical response to BI [13]. While motivation may be a key mediator of behavioral change in BI, investigation on this topic often lack specificity [15, 16]. For example, research is limited in regard to which indices of motivation to change (e.g., importance, confidence, and readiness) facilitate drinking reductions following BI and which individual patient factors moderate response to treatment. Valuable and clinically relevant information may be gained from examining which dimensions of change function as relevant mediators of clinical response as well as identifying patient factors that moderate this response.

Critically, these potential moderators of intervention-based behavior change have only recently begun to be elucidated. Findings have been mixed as to whether BI is appropriate and effective for more severe clinical populations, such as those with comorbid psychiatric conditions or higher alcohol problem severity [17, 18]. Particularly, there is a paucity of research examining the efficacy of BI delivered in medical settings for individuals with very heavy alcohol use or dependence, as these individuals are commonly excluded from randomized trials [18]. However, recent investigations have tested the association between alcohol problem severity and BI utility. For instance, in an emergency department setting, the effects of a therapist-led BI differed across the range of alcohol use severity, such that greater reductions in alcohol consumption were seen for individuals with more severe AUD compared with mild AUD [19]. Additionally, intervention delivery methods, such as computer-based versus in-person counseling, may differentially benefit individuals with lower versus higher severity of alcohol use problems [20]. Low rates of treatment-seeking among individuals with AUD necessitates further work assessing the effectiveness of BI for non-treatment seekers with a range of alcohol problem severity, as BI can be delivered in settings not specialized in substance use treatment [21].

Our group recently conducted a study investigating the efficacy of a brief, single session, intervention for improving drinking outcomes in a non-treatment-seeking heavy drinking sample, which was immediately followed by a neuroimaging scan [22]. Participants reported on their motivation to change drinking behaviors via standardized rulers at baseline and post-intervention timepoints, and 1 month later completed a follow-up visit to assess drinking outcomes. While there was no overall intervention effect on drinking or neural activation to alcohol cues, both the BI and attention-control groups displayed lower drinking rates following participation in the study [22]. Moreover, one dimension of motivation, namely importance of change, was significantly related to neural alcohol cue-reactivity in those who received the BI, as compared to the control condition [23]. These findings advanced our understanding of the neural mechanisms underlying motivation to change elicited by BI but did not account for intervention effects on behavior change in this sample.

The present study extends this work by exploring mechanisms and moderators of behavioral response to BI. In this exploratory analysis, we examined whether participants’ post-intervention motivation to change accounts for treatment response (i.e., drinking reductions) and further, if this response depends on an alcohol problem severity factor. As such, we predicted that intervention-related increases in motivation to change, would result in greater drinking reductions for those with higher alcohol problem severity factor scores. We conducted moderated mediation analyses and hypothesized that the conditional indirect effect of a brief alcohol intervention on drinking outcomes at follow-up through post-intervention motivation to change would be stronger with increasing levels of alcohol problem severity.

## Method

### Participants and screening procedures

This study and procedures were approved by the University of California, Los Angeles Institutional Review Board. Research participants were recruited from the greater Los Angeles metropolitan area via study advertisements describing a research study examining the effects of a brief health education session on beliefs about the risks and benefits of alcohol consumption. The trial was retrospectively registered with clinicaltrials.gov (NCT04710095). Interested individuals completed an initial phone interview and, if determined eligible, were invited into the laboratory for an in-person screening visit. Before study procedures commenced, participants completed the informed consent process and were required to test negative for substances, aside from tetrahydrocannabinol (i.e., THC), on a urine drug test and have a breath alcohol concentration of 0.000 g/dl via a breathalyzer. Participants then completed interviews and a host of individual difference measures.

Individuals were included if they were at least 21 years old and were regular heavy drinkers, as indicated by ≥ 4 drinks consumed per day for females (≥ 5 drinks for males) at least 4 times in the past month and a total Alcohol Use Disorders Identification Test (AUDIT) score of at least 8 points [24]. Individuals were excluded if they were seeking treatment for alcohol use or reported serious alcohol withdrawal symptoms, as determined by a score ≥ 10 on the Clinical Institute Withdrawal Assessment for Alcohol Revised (CIWA-AR) [25]. Full inclusion and exclusion criteria have been previously described, see [22, 23]. In-person screening included 120 individuals, 60 of whom either did not meet inclusion criteria or decided not to participate. Sixty eligible participants were randomized (BI group, n = 32; control group, n = 28) and 52 completed the entire study (BI group, n = 27; control group, n = 25), as eight participants were lost to follow-up. Notably, one participant was excluded from the current analyses for missing 12 days of Timeline FollowBack (TLFB) data, resulting in a final sample of 51 participants. This sample size differs from previous reports on this trial having 46 completers, as usable functional magnetic resonance imaging (fMRI) scan data was a requirement for previous but not current analyses [22, 23].

### Study design

#### Randomization

At the randomization visit, participants completed individual difference measures and were randomly assigned to receive either a one-session BI or an attention-matched control condition, which was facilitated at that visit. Intervention condition was not blinded to research staff or participants. Post-intervention, participants completed a fMRI scan (findings reported previously, see [22]) and self-report measures. Randomized individuals completed a follow-up visit 1 month later to assess changes in alcohol use. Participants who completed all study visits were compensated $160.

#### Brief intervention

The BI consisted of one 30–45 min face-to-face individual session with a therapist based on the principles of motivational interviewing (MI) and adhered to the FRAMES model [26]. The aim of this BI was to help individuals understand their personal level of risk and to help promote and initiate changes in alcohol use. BI sessions were delivered by master’s level clinicians trained in MI techniques (e.g., open-ended questions, reflective listening- seeking understanding of speaker’s experiences and emotional response and offering this back to the speaker, eliciting change talk-language showing desire, consideration, or motivation to change drinking). Every session was audiotaped and rated by a licensed psychologist (author MPK) for fidelity and quality of MI interventions. After intervention sessions, supervision and feedback were provided to therapists by author MPK. Individuals randomized to the attention-matched control condition watched a 30-min video on astronomy. Neither alcohol nor substance use were specifically mentioned in the control condition.

### Measures

#### Clinical interviews

Participants completed the standard TLFB Interview [27] with trained research staff at the initial screening visit and 1-month post-intervention follow-up to assess past month self-reported quantity and frequency of alcohol use, cigarette use, and cannabis use (frequency only). Research staff completed the CIWA-AR [25] interview at the screening visit to assess for symptoms of alcohol withdrawal and the Structured Clinical Interview for DSM-5 [28] to assess for AUD diagnostic criteria and symptom count (possible range 0–11).

#### Individual difference measures

Self-report measures were collected on demographic information, mental health, and substance use patterns. At the screening visit, measures of alcohol problem severity (e.g., hazardous and harmful alcohol use) and tonic craving levels were collected via the AUDIT [24] and Penn Alcohol Craving Scale (PACS; [29]), respectively. To measure participants’ degree of depressive and generalized anxiety symptomatology, well-validated mental health surveys, the Patient Health Questionnaire (PHQ-9) [30, 31] and Generalized Anxiety Disorder 7-Item Scale (GAD-7) [32, 33] were administered.

#### Motivation to change assessment

Participants completed a measure capturing their motivation to change drinking behavior via three motivation to change decision rulers at pre-randomization and immediately post-intervention timepoints. These rulers are used clinically to assess clients’ motivation to change and are described in motivational interviewing protocols and books [34–36]. These rulers display good reliability and predictive validity for drinking and smoking behaviors [37, 38]. On a scale from 1 to 10, participants responded to the following: “As of now how important is it for you to make a change in your drinking?” (*importance ruler*); “If you decided to make a change in your drinking how confident are you that you could do it?” (*confidence ruler*); “As of now how ready are you to make a change in your drinking?” (*readiness ruler*).

### Statistical analysis

All descriptive and statistical analyses were conducted in SAS Version 9.4. Chi-square and independent samples *t*-tests assessed for baseline characteristic differences between the two conditions. Generalized linear models were estimated to present the effect of intervention condition on each motivation to change ruler, covarying for the respective pre-intervention motivation ruler levels. To create a robust alcohol severity score and to reduce the number of variables to be examined in the analyses, a principal component analysis (PCA) with varimax rotation was conducted to create an alcohol problem severity factor across conditions, consistent with previously published studies from our laboratory [39–41]. The PCA included: (a) DSM-5 AUD symptom count, (b) PACS total score, (c) AUDIT total score, (d) PHQ-9 total score, and (e) GAD-7 total score. One factor was retained to represent an alcohol problem severity score (Mean = 0, SD = 1) based on eigenvalues greater than 1 and further examination of the scree plot.

**Fig. 1:**
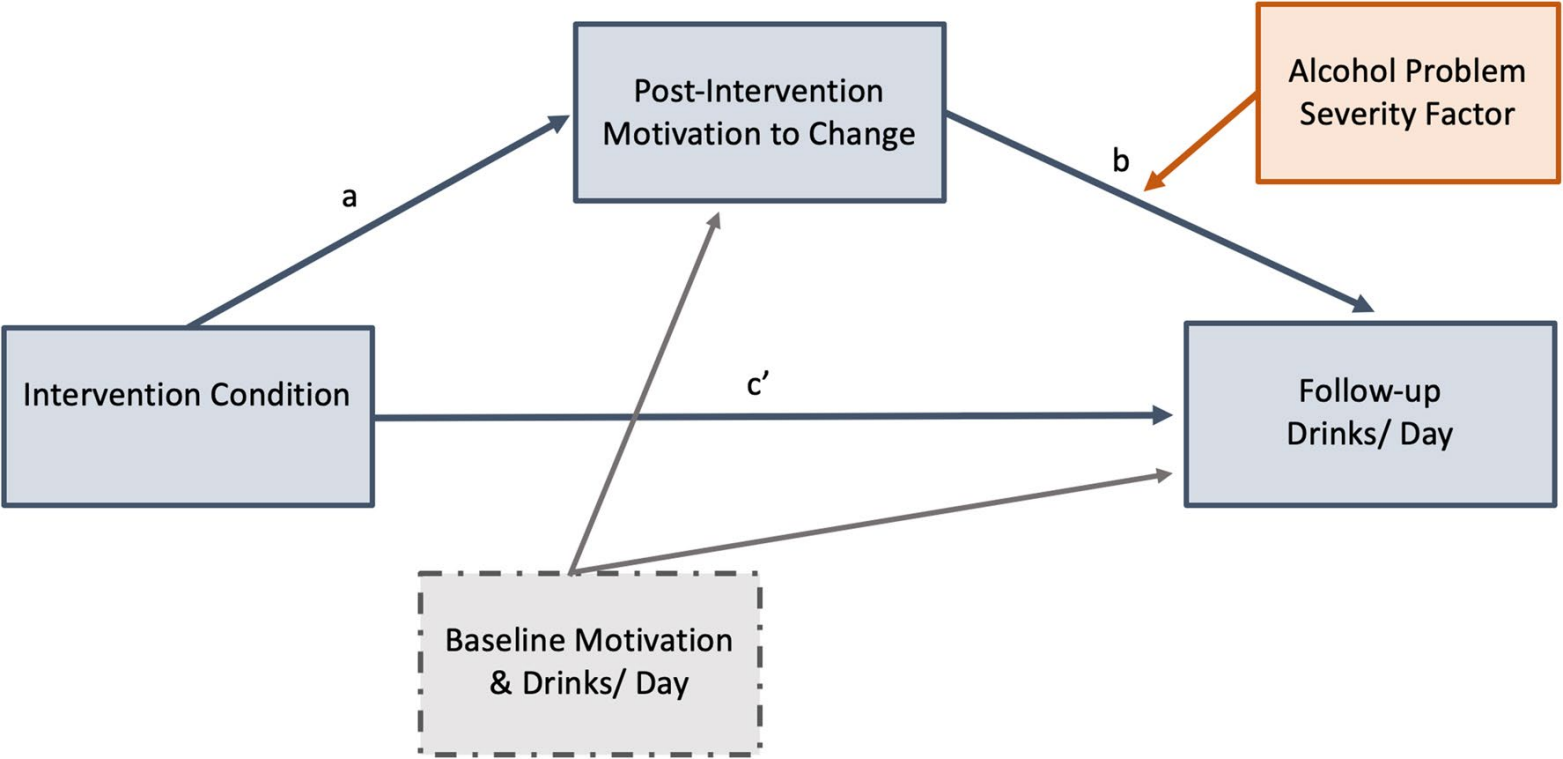
Conceptual diagram for dimensions of motivation to change. Dimensions of motivation to change are importance, readiness, and confidence; intervention condition is a dichotomous variable (0 = control condition; 1 = brief alcohol intervention); alcohol problem severity factor represents participants’ severity factor score from a principal component analysis constructed via baseline measures and interviews

The PROCESS macro (Model 14) was used to conduct three conditional process analyses (i.e., moderated mediation; [42]). Consistent with a previous report on this trial [23], separate models were run for each of the three post-intervention motivation to change rulers (i.e., importance, confidence, and readiness). As such, the effect of intervention (dichotomous focal predictor; 0 = control condition, 1 = brief alcohol intervention) on follow-up average alcoholic drinks per day (continuous outcome variable) through motivation to change (continuous mediator) were examined (see Fig. 1). Direct effects and conditional indirect effects were tested. The alcohol problem severity factor (continuous) was added in the model as a second-stage moderator, as we hypothesized that the effect of motivation on drinking behaviors would be stronger for those with higher problematic alcohol use. To capture a broad range of problem severity, both the interaction term and conditional indirect effects were probed at alcohol problem severity factor score values of − 0.85, − 0.29, and 1.10, which correspond to the 16th, 50th, and 84th percentile of total sample severity scores (i.e., default PROCESS macro percentile values). Baseline alcoholic drinks per day and the corresponding baseline motivation to change score were added to the model as covariates. Percentile bootstrap confidence intervals were estimated for the indices of moderated mediation (k = 15,000). Partial missing data existed for three participants on the follow-up TLFB interview (1–2 days missing out of 30 days) for which mean imputation was used.

## Results

### Participant characteristics

The final sample consisted of 51 non-treatment-seeking heavy drinking adults (BI group, n = 27; control group, n = 24) reporting problematic alcohol use who completed all study assessments, including the 1-month follow-up visit (85% of randomized participants). The sample was 57% male with an average age of 35 years. Participants endorsed an average of 4 DSM-5 AUD symptoms, corresponding to moderate AUD on average. Individuals randomized to BI as compared to the control condition did not differ significantly on a range of baseline characteristic variables (see Table 1). Scores for motivation to change rulers at pre- and post-intervention timepoints are reported in Table 2. Pre-intervention motivation to change scores did not significantly differ between intervention conditions (*p*’s > 0.20). At post-intervention, only importance of change scores differed significantly between conditions (*p* < 0.002), after covarying for pre-intervention motivation to change levels.

**Table 1:** Baseline sample characteristics of participants by intervention condition

Variable Mean (standard deviation)	Total sample (N = 51)	Brief intervention group (n = 27)	Control group (n = 24)	Sign. (*p*-value)
Age (years)	34.6 (12.4)	35.3 (13.7)	33.9 (11.0)	0.680
Sex (% male)	56.9%	55.6%	58.3%	0.842
Education (years)	15.1 (1.9)	15.3 (2.0)	15.0 (1.8)	0.528
Drinks per day (past month)	3.3 (1.8)	3.5 (2.6)	3.2 (1.7)	0.573
Drinks per drinking day (past month)	5.5 (2.0)	5.5 (2.3)	5.5 (1.6)	0.878
% Days abstinent (past month)	40.7% (23.8)	37.4% (25.7)	44.3% (21.3)	0.306
DSM-5 AUD symptoms (total count)	4.1 (2.5)	4.1 (2.5)	4.1 (2.7)	0.985
Alcohol Use Disorders Identification Test (total score)	17.5 (7.4)	17.6 (7.2)	17.5 (7.7)	0.935
Penn Alcohol Craving Scale (total score)	19.8 (6.6)	19.9 (6.7)	19.6 (6.7)	0.874
% Cigarette use (past month)	45.1%	44.4%	45.8%	0.921
% Positive THC screen (urine drug test)	27.5%	30.0%	25.0%	0.712
Patient Health Questionnaire-9 (total score)	5.4 (5.1)	5.3 (5.1)	5.4 (5.3)	0.977
Generalized Anxiety Disorder 7-Item Scale (total score)	4.0 (5.0)	3.7 (5.0)	4.4 (5.2)	0.620

A heavy drinking day is defined as ≥ 4 drinks consumed for females or ≥ 5 drinks for males; alcohol and cigarette use variables were determined using past-month Timeline FollowBack interview; AUD symptom count assessed via the Structured Clinical Interview for DSM-5; chi-square or independent samples *t*-tests assessed for baseline characteristic differences between conditions

**Table 2:** Motivation to change ruler scores by intervention condition and timepoint

Variable Mean (standard deviation)	Total sample (N = 51)	Brief intervention group (n = 27)	Control group (n = 24)	Sign. (*p*-value)
Pre-intervention^a^				
Importance ruler	4.8 (2.6)	4.4 (2.5)	5.3 (2.8)	0.216
Confidence ruler	5.9 (2.6)	5.6 (2.8)	6.1 (2.5)	0.509
Readiness ruler	3.5 (2.0)	3.3 (2.0)	3.8 (2.1)	0.321
Post-intervention^b^				
Importance ruler	5.5 (2.3)	6.0 (2.2)	5.0 (2.4)	0.001*
Confidence ruler	6.8 (2.4)	7.1 (2.4)	6.5 (2.5)	0.169
Readiness ruler	4.8 (2.3)	4.8 (2.4)	4.8 (2.3)	0.561

Motivation to change ruler scores can range from 1 to 10 points

^a^Independent samples *t*-test assessed pre-intervention motivation to change differences between conditions

^b^Generalized linear models assessed post-intervention motivation to change differences by condition, covarying for pre-intervention scores

*Indicates significance at the *p* < 0.05 level

### Principal component analysis for alcohol problem severity factor

The PCA of DSM-5 AUD symptom count and PACS, AUDIT, PHQ-9, and GAD-7 total scores, yielded one factor with all variables loading > 0.40 on the alcohol problem severity factor. The PCA explained approximately 68% of the total variance (Eigenvalue = 3.387; see Table 3). This supports the use of the alcohol problem severity factor score in subsequent analyses.

**Table 3:** Principal component analysis factor loadings for alcohol problem severity

Variable	Alcohol problem severity factor
Penn Alcohol Craving Scale total	0.71
DSM-5 SCID AUD symptom count	0.85
Alcohol Use Disorders Identification Test total	0.82
Patient Health Questionnaire-9 total	0.90
Generalized Anxiety Disorder 7-Item Scale total	0.82

Principal component analysis yielded one factor with all variables loading > 0.40 that explained 68% of the total variance (Eigenvalue = 3.387)

### Primary findings

#### Importance of change ruler

**Fig. 2:**
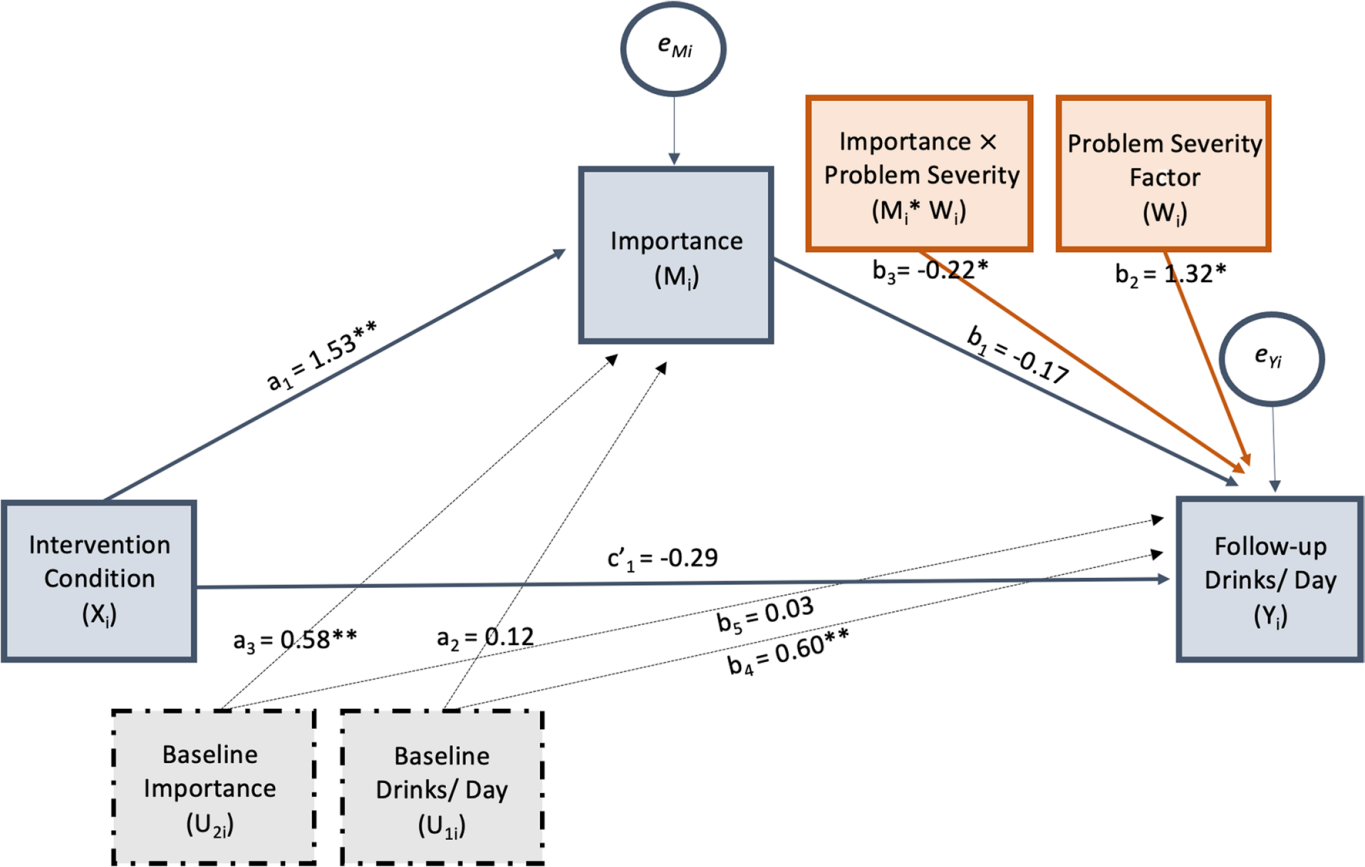
Statistical diagram and model equations for importance of change conditional process analysis. Conditional process model equations: $Y_{i} = c^{\prime}_{0} + c^{\prime}_{1} X_{i} + b_{1} M_{i} + b_{2} M_{i} + b_{3} M_{i} *W_{i} + b_{4} U_{1i} + b_{5} U_{2i} + e_{yi}$_;_ $M_{i} = a_{0} + a_{1} X_{i} + a_{2} U_{1i} + a_{c} U_{21} + e_{Mi}$_;_ $Y_{i} = 1.43 - 0.29X_{i} - 0.17M_{i} + 1.32W_{i} - 0.22M_{i} *W_{i} + 0.60U_{1i} + 0.03U_{2i} + e_{yi}$_;_ $M_{i} = 1.52 + 1.53X_{i} + 0.12U_{1i} + 0.58U_{2i} + e_{Mi}$. Intervention condition is a dichotomous variable (0 = control condition; 1 = brief alcohol intervention); alcohol problem severity factor represents participants’ severity factor score from a principal component analysis constructed via baseline measures and interviews; baseline importance of change and baseline drinks per day served as covariates; * indicates significance at the *p* < 0.05 level and ** at the *p* < 0.01 level

The direct effect of condition on follow-up alcoholic drinks per day was not significant, $c^{\prime}_{1}$ = − 0.30 (*t*(44) = − 0.75, *p* = 0.459) when holding all other predictors and covariates constant (see Fig. 2). The *a* path (effect of condition on importance of change) was significant, *a*_1_ = 1.53 (*t*(47) = 3.23, *p* < 0.003) after accounting for covariates with a model *R*^2^ = 0.53. For the *b* path (model *R*^2^ = 0.61), the conditional effect of importance on follow-up drinks per day, was not significant (*p* = 0.132), but the interaction term was significant, *b*_3_ = − 0.22 (*t*(44) = − 2.50, *p* = 0.016, after holding condition and baseline covariates constant. When probing this interaction term using a simple-slopes technique at the 16th, 50th, and 84th percentiles, results indicated that only at the highest problem severity percentile probed was the conditional effect of importance on follow-up drinks per day significant, *b* = − 0.41, (*t*(44) = − 2.62, *p* = 0.012). Importantly, this conditional process analysis yielded a significant index of moderated mediation, *a*_1_*b*_3_ = − 0.33 (95% CI − 0.79, − 0.02), suggesting that the effect of the BI on follow-up drinks per day through importance of change was moderated by alcohol problem severity level after accounting for baseline covariates. This indirect effect of BI condition on follow-up drinks per day was probed at the same values of the moderator using a pick-a-point approach. Results indicated that the bootstrapped confidence interval for the indirect effect of intervention condition on drinking through importance was again only significant at the highest severity percentile probed (95% CI − 1.34, − 0.005; see Table 4). Considering effect direction, findings suggest that among individuals with high problem severity, receiving the BI as compared with the control condition, was predictive of lower drinks per day at follow-up through increases in importance to change ratings.

**Table 4:** Conditional indirect effects of intervention condition on alcohol use through motivation to change at probed alcohol problem severity factor values

Alcohol problem severity factor value (percentile)	Effect b (standard error)	Bootstrap 95% confidence interval (range)
Importance		
− 0.85 (16th percentile)	0.02 (0.20)	− 0.33, 0.51
− 0.29 (50th percentile)	− 0.16 (0.18)	− 0.51, 0.21
1.10 (84th percentile)	− 0.63 (0.30)	− 1.34, − 0.005*
Confidence		
− 0.85 (16th percentile)	0.05 (0.12)	− 0.26, 0.23
− 0.29 (50th percentile)	− 0.01 (0.10)	− 0.30, 0.13
1.10 (84th percentile)	− 0.16 (0.19)	− 0.59, 0.18
Readiness		
− 0.85 (16th percentile)	− 0.003 (0.06)	− 0.12, 0.12
− 0.29 (50th percentile)	− 0.03 (0.07)	− 0.20, 0.09
1.10 (84th percentile)	− 0.08 (0.19)	− 0.58, 0.21

Moderator was probed at the 16th, 50th, and 84th total sample percentile of alcohol problem severity factor using the pick-a-point approach to determine the conditional indirect effect of intervention condition on follow-up drinks per day through changes in motivation after accounting for corresponding baseline motivation and drinking; beta estimates are unstandardized

*Denotes significant conditional indirect effect

#### Confidence in change ruler

**Fig. 3:**
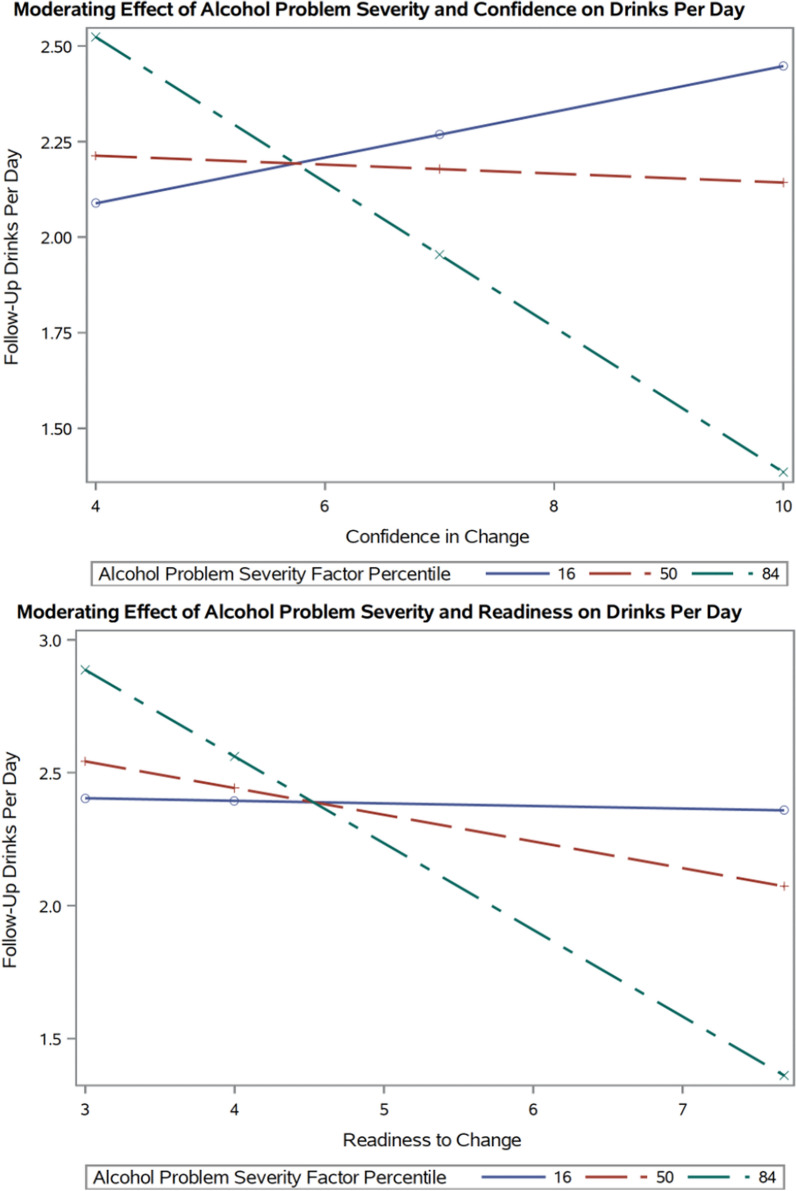
Moderating effect of alcohol problem severity factor on the relationship between motivation to change indices and follow-up drinks per day. This depiction shows a significant second stage interaction effect of alcohol problem severity factor by post-intervention motivation to change indices on follow-up drinks per day across intervention conditions and after holding baseline ratings and drinks per day constant; alcohol problem severity represents participants’ severity factor score from a principal component analysis constructed via baseline measures and interviews; the interaction effects are presented at the probed 16th, 50th, and 84th percentile alcohol severity factor values for the total sample

Similarly, the direct effect of condition on follow-up alcoholic drinks per day was not significant, $c^{\prime}_{1}$ = − 0.39 (*t*(44) = − 1.07, *p* = 0.290), when holding all other predictors and covariates constant. The *a* path was also not significant, *a*_1_ = 0.85 (*t*(47) = 1.41, *p* = 0.167) after accounting for covariates with a model *R*^2^ = 0.26. For the *b* path (model *R*^2^ = 0.59), the conditional effect of confidence on follow-up drinks per day was not significant (*p* = 0.582). However, the interaction term was significant, *b*_3_ = − 0.13 (*t*(44) = − 2.15, *p* = 0.037), after holding condition and baseline covariates constant (see Fig. 3), but none of the individual simple slopes probed were significant (*p*’s > 0.05). The conditional process analysis did not yield a significant index of moderated mediation, (95% CI − 0.31, 0.14), such that we did not detect a significant moderating effect of the alcohol problem severity factor on the indirect effect of BI on drinking at follow-up through confidence. The indirect effect of BI condition on follow-up drinks per day was probed at the same values of the moderator using a pick-a-point approach. Results indicated that the bootstrapped confidence interval for the indirect effect of intervention condition on drinking through confidence was not significant at any of the three severity percentiles probed (95% confidence intervals contain 0; see Table 4).

#### Readiness to change ruler

The direct effect of condition on follow-up alcoholic drinks per day was not significant, $c^{\prime}_{1}$ = − 0.51 (*t*(44) = − 1.54, *p* = 0.130), when holding all other predictors and covariates constant. The *a* path was not significant, *a*_1_ = 0.25 (*t*(47) = 0.46, *p* = 0.651), after accounting for covariates with a model *R*^2^ = 0.31. For the *b* path (model *R*^2^ = 0.66), the conditional effect of readiness on follow-up drinks per day was not significant (*p* = 0.107). However, the interaction term was again significant, *b*_3_ = − 0.16 (*t*(44) = − 2.10, *p* = 0.042), after holding condition and baseline covariates constant and the highest severity factor percentage (84th percentile) probed using simple slopes was significant (*p* = 0.005; see Fig. 3). The conditional process analysis did not yield a significant index of moderated mediation, (95% CI − 0.31, 0.12), such that we did not detect a significant moderating effect of the alcohol problem severity factor on the indirect effect of BI on drinking at follow-up through readiness. Results indicated that the bootstrapped confidence interval for the indirect effect of intervention condition on drinking through readiness was not significant at any of the three severity percentiles probed (95% confidence intervals contain 0; see Table 4).

## Discussion

This study explored both mechanisms and moderators of behavioral response to BI in a sample of non-treatment seeking heavy drinkers. Overall, our hypothesis regarding the indirect effect of intervention on drinking at follow-up was supported for only one dimension of motivation to change. Specifically, the effect of BI on drinks per day at follow-up through enhancing importance for behavior change was stronger for those with high alcohol problem severity factor scores. Our preliminary results indicate that for individuals with higher degree of problem severity, the BI significantly reduced alcohol use at follow-up through the mechanism of promoting participants’ importance to make a drinking behavior change. Thus, it appears that as severity increased in this sample, the therapeutic effect of importance on post-intervention alcohol use became stronger. These effects were significant while controlling for baseline covariates of drinks per day and importance of change rating. Findings suggest that severity may be a relevant factor to consider in regard to the efficacy of BI in non-treatment seeking populations. Yet, this preliminary finding should be interpreted with caution and replicated in larger samples of brief alcohol intervention. Results should also be considered in the context of this study’s sample, which included heavy drinkers not required to meet DSM-5 criteria for AUD. As such, it represents a low- to moderate-severity sample overall. Our results are in line with recent findings examining the role of problem severity in the context of brief alcohol interventions [19, 20]. As reported previously by our group, this BI did not significantly reduce drinking more than the control condition [23]. Although not assessed in the current study, BIs are thought to be well-suited as an initial treatment contact for non-treatment seeking individuals with risky alcohol use that may prepare them to engage in later specialty treatment. A secondary analysis of data from the National Survey on Drug Use and Health found that only around 16% of individuals who received information about alcohol treatment from their healthcare provider obtained it [21], signaling the potential benefit of providing BI to these individuals, as it may increase one’s motivation to seek further alcohol treatment or serve as a limited opportunity for intervention [43, 44].

Markedly, in the second stage of mediation models for all three indices of motivation to change (i.e., importance, readiness, and confidence), the interaction term between post-intervention motivation and alcohol problem severity factor was significant after holding treatment condition and other covariates constant. Both groups completed alcohol-specific assessments and interviews at the baseline visit and this likely increased participants’ awareness of their drinking levels and related consequences [45], which in turn could have increased participants’ motivation for behavior change following the baseline visit across groups. In line with our results, one’s self-identified motivation to change drinking following a clinical contact may be a possible indicator of future drinking reductions for those with elevated problem severity, irrespective of intervention-specific effects. As such, even a general screening for problematic or heavy alcohol use in medical settings might provide small benefits to individuals with at least moderate severity levels. Alternatively, this finding could point to a potential mechanism of ‘natural recovery’ for individuals high in alcohol problem severity, in which life events and consequences result in an increase in their motivation to reduce alcohol use, which in turn leads to cutting back on drinking [46, 47]. However, these interpretations are speculative and should be carefully tested in larger samples. Notably, the alcohol problem severity factor used in these analyses included measures of mental health symptoms (i.e., generalized anxiety and depression symptomatology) in addition to alcohol-specific measures, which is consistent with a previous report from our group [40]. Prior work highlights the relevance of negative affectivity in clinical AUD samples, as it is positively correlated with severity, such that those with more severe AUD report higher levels of negative affectivity [48, 49].

Findings from the first path of the importance mediation model suggested that participants receiving the BI reported significantly greater importance of change ratings compared to those in the control condition after accounting for pre-intervention importance levels. Accordingly, this dimension of motivation to change may serve as a mechanism of the BI implemented in this study. Importance of change can be considered the first tier or step towards increasing one’s motivation to change, akin to the contemplation stage of change (i.e., individual acknowledges risky drinking and may be open to change but remains ambivalent; [7]). The role of this initial stage of motivational readiness in this BI effect may be especially salient for the study’s population of community-based heavy drinking individuals who are not considering treatment. It should also be noted that single-item ratings of importance to change correlate highly with a stage-based multi-item measure of motivational readiness [50]. Hence, the current findings may actually speak more broadly about the role of stage of change as a mechanism of BI in non-treatment seekers. However, treatment mechanisms may vary across brief alcohol interventions, as clinician training and implementation of BIs can differ, and as a result may contain distinctive treatment components. Further research aimed at better understanding which components of BI promote one’s motivation to change is warranted.

Intervention-related alterations in readiness and confidence dimensions of motivation to change were not detected in this study. This modest sample size lacks statistical power to detect small effect sizes, and this may have contributed to our null findings. Results may also point to issues that may be particularly relevant to the delivery of BI to non-treatment seeking individuals. A single-session brief behavioral intervention may be an insufficient dose to yield measurable changes in confidence or readiness to change among individuals who are not already motivated to change their alcohol use. Extant research on BI with non-treatment seeking populations (e.g., patients in the emergency department and college students) has also not found consistent support for a mediating role of readiness to change [51, 52]. Thus, our findings only support importance as a potential mechanism of behavior change in this sample, particularly for individuals with high severity. Future research might explore whether more time-intensive interventions (e.g., Motivational Enhancement Therapy) are necessary to engender changes in one’s feelings of readiness or confidence to make drinking-related changes and whether this might be specific to treatment-seeking populations. Alternatively, these indices of motivation may similarly function as mechanisms of behavioral change, but BIs may provoke smaller changes in readiness and confidence motivational indices than importance, thus requiring larger or higher severity samples to detect these effects.

The current study should be considered in light of its strengths and limitations. First, this conditional process analysis consisted of variables collected with temporal precedence, which provides partial supports, albeit not sufficient evidence, for these mechanisms as causal. Second, the advanced statistical methods applied (i.e., conditional process analysis) are well-suited for examining complex relationships between mechanisms and subgroups of responders for whom these mechanisms are operative. Third, participants were recruited from the community and displayed a range of alcohol problem severity allowing for examination of our multi-method severity factor construct as a moderator. In terms of limitations, the current analyses relied on self-report measures of alcohol consumption, which can be susceptible to bias and drink size misestimation, resulting in possible inaccuracies in intervention-related outcomes [53]. Moreover, the sample size is modest, and this study is not powered to detect small effects in mediation pathways. As such, while our results provide initial support for certain moderation and mediation effects, the analyses were exploratory and should be interpreted with caution and replicated in larger trials of brief alcohol intervention. Neither participants nor study staff were blinded to treatment condition after randomization, which could have contributed to social desirability bias in the treatment effect [54, 55]. Participants rated their motivation to change drinking behaviors immediately following the brief intervention; this limited timeframe could have failed to capture more long-term intervention-related changes in motivation, particularly in the confidence and readiness dimensions. Finally, while participants reduced their drinking levels from baseline to follow-up regardless of treatment condition, the intervention main effect was not detected in this study.

## Conclusion

On balance, this exploratory investigation adds to the literature on BI for alcohol problems in non-treatment seeking samples and suggests that brief alcohol interventions may be more impactful for individuals with higher levels of alcohol problem severity, particularly through intervention-driven changes in ratings of importance of behavior change. Additionally, findings highlight the potential relevance of considering participants’ level of severity, even as it pertains to non-intervention specific alcohol assessment/screening factors, which might promote one’s motivational levels and reduce drinking among particular individuals, although further research on this topic is necessary. Future studies that continue to integrate neural and behavioral levels of analysis to explore mechanisms of behavior change are needed to more fully elucidate markers of response to BI for hazardous drinking.

## Data Availability

The datasets used and/or analyzed during the current study are available from the corresponding author on reasonable request.

## Abbreviations

BI: Brief intervention
AUD: Alcohol use disorder
AUDIT: Alcohol Use Disorders Identification Test
CIWA-AR: Clinical Institute for Withdrawal Assessment for Alcohol Revised
TLFB: Timeline FollowBack
fMRI: Functional magnetic resonance imaging
MI: Motivational interviewing
PACS: Penn Alcohol Craving Scale
PHQ-9: Patient Health Questionnaire-9
GAD-7: Generalized Anxiety Disorder 7-Item Scale
PCA: Principal component analysis
